# The efficacy of Enhanced Recovery after Surgery (ERAS) for elderly patients with intertrochanteric fractures who received surgery: study protocol for a randomized, blinded, controlled trial

**DOI:** 10.1186/s13018-020-01586-w

**Published:** 2020-03-05

**Authors:** Mengchen Yin, Yinjie Yan, Zhaoxiang Fan, Niankang Fang, Hongbo Wan, Wen Mo, Xuequn Wu

**Affiliations:** Department of Orthopaedics, LongHua Hospital, Shanghai University of Traditional Chinese Medicine, Shanghai, China

**Keywords:** Intertrochanteric fracture, Enhanced recovery after surgery, Perioperative period, Integrating TCM with and western medicine, Randomized controlled trial

## Abstract

**Background:**

Intertrochanteric fracture (ITF) is increasing with the rapid increase in the aging population, often causes a high mortality rate in old patients and increases the economic burden of the family and society. ERAS (Enhanced Recovery after Surgery) is a powerful guarantee for patients to accelerate their recovery after surgery. TCM (traditional Chinese medicine) promotes repair of injured tissues and eliminates traumatic aseptic inflammation. Therefore, this prospective randomized controlled clinical trial aims to evaluate the clinical effect of the evidence-based ERAS pathway of integrating TCM with western medicine on perioperative outcomes in ITF patients undergoing intramedullary fixation and provide reliable evidence-based data for applying the program to clinical practice.

**Methods/design:**

We will conduct a prospective randomized, blinded, controlled trial to compare the effectiveness of ERAS care pathway with traditional care pathway and to investigate whether the ERAS care pathway can improve the perioperative outcome in ITF patients undergoing intramedullary fixation. A total of 60 patients with ITF will be enrolled and treated with the two care pathway, respectively. Length of stay, economic indicators, Harris score, VAS score, time to get out of bed, 30-day readmission rates, postoperative transfusion rates, discharge to home, and mortality will be evaluated. Any signs of acute adverse reactions will be recorded at each visit during treatment.

**Discussion:**

Although an evidence-based process using the best available literature and Delphi expert-opinion method has been used to establish an ERAS pathway of integrating TCM with western medicine, there is a lack of consensus about its effectiveness. This trial will provide convincing evidence about the effect of ERAS pathway.

**Trial registration:**

Registered on 12 October 2019. Trial number is ChiCTR1900026487.

## Introduction

With the aging of society, the incidence of intertrochanteric fracture (ITF) is increasing. It is easy to bring bedsore, urinary tract infection, lung infection, and other complications. Intertrochanteric fracture has become a major public health issue with a high mortality rate for old patients and increased the economic burden [1, 2]. Currently, ITF is treated with both surgical and non-surgical methods. Surgical treatment has great advantages in alleviating pain, restoring hip function, improving quality of life, and avoiding complications such as deep vein thrombosis and cardiovascular accident caused by long-term bed rest, so surgical treatment is the first choice of treatment [3–6]. Currently, PFNA (proximal femoral nail anti-rotation, PFNA) is a mature surgical treatment for senile ITF, which is characterized by maintaining strong fixation, biomechanical stability, and minimal invasion [7, 8].

ERAS (Enhanced Recovery after Surgery) is one of the two important development directions leading the development of modern surgery in the twenty-first century, which is first proposed by Kehlet in 1997 [9]. ERAS aims to integrate the perioperative diagnosis and treatment concept by optimizing a series of effective approaches and methods with evidence-based medical evidence, so as to reduce trauma invasion and alleviate the stress reaction caused by surgery. The core of the ERAS is a powerful guarantee for patients to accelerate their recovery after surgery [10–13]. At present, ERAS has been applied in the treatment of many orthopedic diseases including artificial joint replacement [14].

Currently, major efforts are being conducted to transfer the lessons learned from other surgical specialties and incorporate ERAS protocols into postoperative care of patients undergoing hip surgery. ERAS protocols for artificial joint replacement have been described that it can reduce postoperative mortality and increase satisfaction for patients. However, to our best knowledge, ERAS programs for patients with ITF have not been described. Furthermore, traditional Chinese medicine including acupuncture, manipulative therapy, and oral Chinese medicine can improve blood circulation in local tissues, relax tense and spasmodic muscles, promote repair of injured tissues, and eliminate traumatic aseptic inflammation. Therefore, it is of great significance to establish an effective plan and management pathway integrated Chinese and western medicine to accelerate postoperative recovery of ITF. With the objective in mind, we have conducted a systematic literature search to guarantee the comprehensiveness of the study; Delphi expert-opinion method was used to establish the ERAS pathway. Here, ERAS programs consist of a multidisciplinary evidence-based approach to preoperative management, intraoperative control, and rehabilitation for ITF, and preoperative multidisciplinary collaboration had been already established.

Therefore, this prospective, randomized, controlled clinical trial aims to evaluate the clinical effect of an evidence-based ERAS pathway integrated Chinese and western medicine on perioperative outcomes in ITF patients undergoing intramedullary fixation and provide reliable evidence-based data.

## Materials and methods

### Study design

This study is a prospective, outcome assessor- and data analyst-blinded, randomized controlled design study that compared an ERAS care pathway cohort with standardized care pathway cohort. Emergency admissions with a primary diagnosis of ITF between January 2020 and July 2021 in our hospital will be selectively enrolled. All patients will receive their operation at our hospital where elective surgeries take place predominantly, and they will be treated with PFNA intramedullary fixation at our institution.

**Fig. 1:**
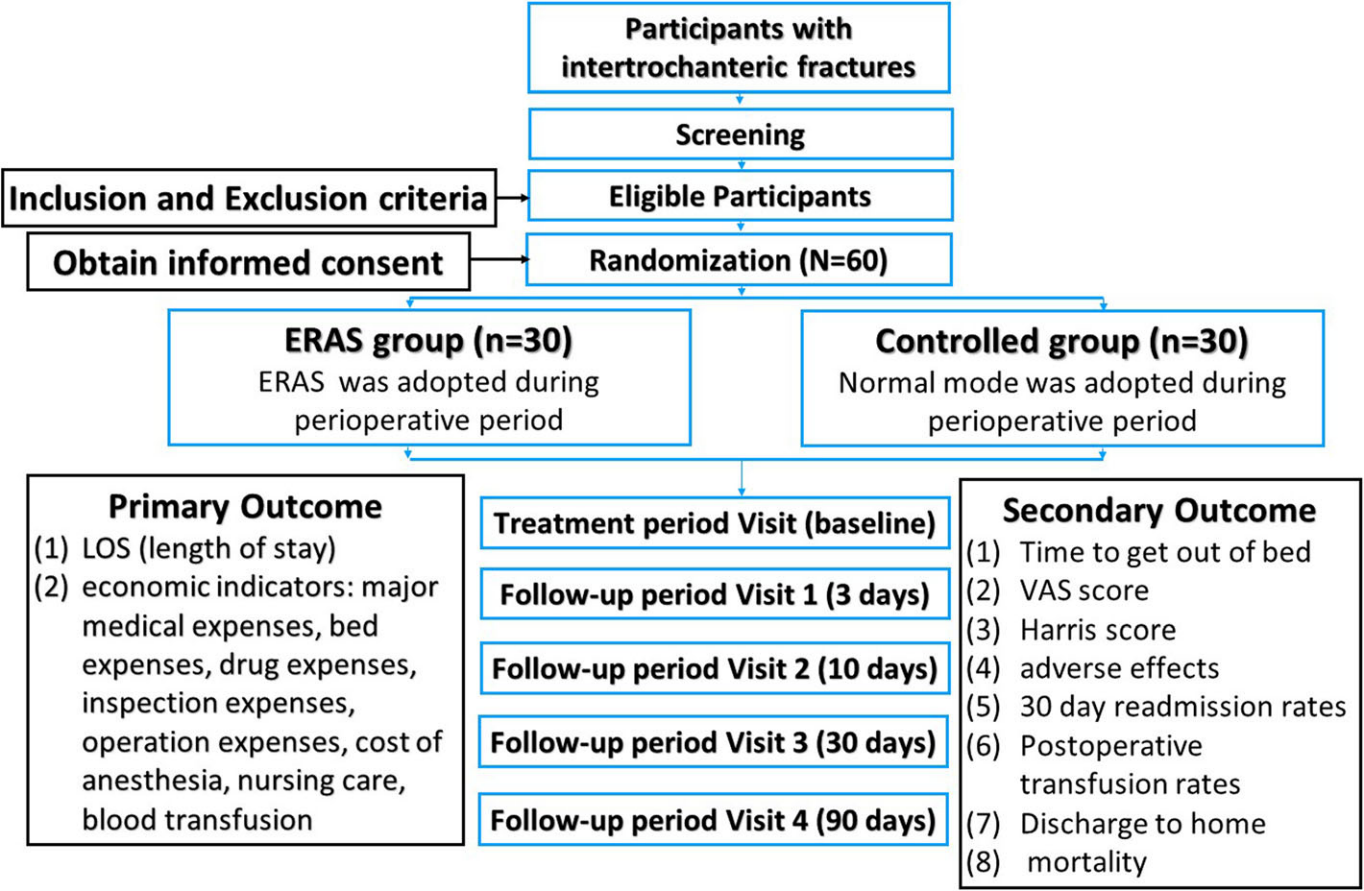
Participant processing and the schedule of evaluation

The objective of this proposed study is to investigate whether ERAS care pathway will lead to perioperative improvement in ITF patients undergoing intramedullary fixation. The principal investigator (PI) is responsible for the overall project and organizing Steering Committee meetings. PIs of sub-center departments are responsible for gathering experts to carry out the project. An independent Steering Committee will be responsible for affairs such as participants’ safety, meetings, recruitment and follow-up of participants, and quality control. The coordinating center is responsible for communicating protocol modifications and providing materials. This trial includes a 2-week treatment period and a 3-month follow-up period. Outcome assessments will be conducted at baseline, as well as at 3, 14, 30, and 90 days (Fig. 1).

### Eligibility criteria

#### Inclusion criteria

Participants who meet the criteria below are eligible [15]:

- The patients are over 75 years old.
- X-ray or CT indicates unstable ITF.
- Treated with PFNA intramedullary fixation.
- Being willing to undergo surgery with the ERAS pathway.
- Being willing to give informed consent.

#### Exclusion criteria

The exclusion criteria are as follows [15]:

- Patients are with open fractures or pathological fracture caused by tumor, infection, or tuberculosis.
- Patients are with congenital hip dysplasia or osteonecrosis of the femoral head.
- Hip surgery before enrollment.
- Failure to understand or sign informed consent.

### Patient population and recruitment procedure

Participants will be recruited from Longhua Hospital affiliated to Shanghai University of Traditional Chinese Medicine. Prospective participants will be interviewed by the coordinators and informed of the eligibility criteria and the procedure. Those who are eligible and willing to participate in the study will be screened initially by baseline assessment and then diagnosed based upon clinical manifestations, physical examination, and imaging. Participants will be informed that participating in the trial was absolutely voluntary and withdrawal from the trial can be made at any time. In case of withdrawal, the data collected will not be deleted and will be used in the final analyses. A data compilation form including all variables of interest and all potential risks will be completed by the corresponding research center. The information obtained will be stored in an electronic database for subsequent statistical analysis. Recruitment will start in January 2020 and is expected to end in July 2021. The final follow-up of all participants will be completed on 30 September 2021. The overview of the participant processing and the schedule of evaluation is provided in Fig. 1.

### Patient and public involvement

Patients and/or the public will not be involved in the study design and study enrolment.

### Ethics

This study is to be conducted in accordance with the principles of the Declaration of Helsinki and has been approved by Sichuan Regional Ethics Review Committee on Traditional Chinese Medicine (TCM) [16]. The trials have been approved by the appropriate Institutional Review Boards. The clinical trial has been approved by the Institutional Review Board of Longhua Hospital, Shanghai University of TCM. All participants will be given sufficient time to reach a decision to sign the consent form prior to the study in compliance with Good Clinical Practice Guidelines that guides the appropriate use of TCM in clinical practice. Then, the participants will be randomized into two groups of different treatments. Reporting will be guided by the CONSORT statement [17, 18].

### Intervention

#### ERAS care pathway

An evidence-based process using the best available literature and Delphi expert-opinion method was used to establish the ERAS pathway integrated Chinese and western medicine. The basic components of the multi-disciplinary ERAS pathway which we used are shown in Table 1. The principles include educational program, management of nutrition, management of dietary, management of sleep, management of pain selection of anesthesia, control of bleeding, management of body temperature, prevention of infection, management of anesthesia, management of rehydration management of drainage tube, control of nausea and vomiting, and management of activity.

**Table 1:** The basic components of the multi-disciplinary ERAS pathway

Preoperative	Educational program	(1) Understand the patient, assess the condition
		(2) Psychological, nutrition, surgery, rehabilitation education
		(3) Good communication
		(4) Emphasize active function exercise
		(5) Advocate deep breathing, upper limbs pull rings, and other cardiopulmonary exercise
	Management of nutrition	(1) If there is hypoalbuminemia and severe anemia, actively look for the original disease and correct it
		(2) When necessary, human serum albumin 10 g Ivgtt
		(3) Megaloblyte anemia: folate 5–10 mg Po Tid+ vitamin B12 0.5 mg Im Tiw
		(4) Iron deficiency anemia: EPO 10,000 IU Ih Tiw+ ferrous succinate 0.2 g Po Tid
	Management of dietary	(1) Eat a high protein diet
		(2) Before anesthesia 6 h fast protein liquid (such as milk, broth)
		(3) Before anesthesia 4 h fast carbohydrates (such as rice porridge, steamed bread)
		(4) 2 h before anesthesia, do not drink clear liquid
		(5) When necessary, 250–500 ml glucose was dropped 2–3 h before operation
	Management of sleep	(1) Sedative hypnotic or anti-anxiety drugs
	Management of pain	(1) Routine use of anti-inflammatory analgesics such as celecoxib 200 mg Po Bid
Intraoperative	Selection of anesthesia	(1) General anesthesia (laryngeal mask or endotracheal intubation)
		(2) Combined with local infiltration anesthesia: ropivacaine 200 mg + 80 ml saline was injected into the incision and surrounding deep needle
	Control of bleeding	(1) Blood pressure control: systolic blood pressure control in 90–110mmhg
		(2) Bleeding control: 5–10 min before skin incision, tranexamic acid should be dropped 15–20 mg/kg
	Management of body temperature	(1) Monitor and dynamically adjust the operating room temperature, do a good job of keeping warm
		(2) Reduce limb exposure, for patients covered inflatable heating blanket
		(3) The infusion of liquid will be first heated to 37 °C
	Prevention of infection	(1) Ensure the operating room environment, control the number of patients involved in the operation
		(2) Strict disinfection towel, as far as possible to shorten the operation time and reduce the surgical trauma, the operation field repeatedly rinse
		(3) Preoperative 0.5–2 h intravenous antibiotics
		(4) If the operation time exceeds 3 h, or blood loss > 1500 ml with the second dose
		(5) The effective coverage time of antibacterial drugs includes the whole surgical process and 4 h after surgery, and the total prevention time is no more than 24 h
Postoperative	Management of anesthesia	(1) General anesthesia wake up: drink water before eating
		(2) Moxapride 5 mg Po Tid to improve gastrointestinal motility
		(3) Selection of anesthesia
	Management of rehydration	(1) Avoid a large amount of fluid replacement: infusion volume from 25 to 40 ml (kg/day) is appropriate
		(2) Control the infusion speed: the infusion speed of elderly patients is from 100 to 120 ml/h is appropriate
		(3) Monitor blood routine, liver function, kidney function, and cardiac function indicators
	Management of drainage tube	(1) No drainage or catheter was placed
	Control of nausea and vomiting	(1) Intraoperative intravenous use of dexamethasone 10 mg
		(2) Use ondansetron when necessary
	Management of sleep	(1) Sedative hypnotic or anti-anxiety drugs
	Management of pain	(1) Use of automatic analgesia pump for 3 days
		(2) Sequential use of anti-inflammatory and analgesic drugs, such as celecoxib 200 mg Po Bid (recommended reduction of 50% for liver damage and elderly patients)
	Management of activity	(1) Emphasis on early hip, knee, and ankle active flexion and extension function exercise, to increase muscle strength
		(2) Exercise passive joint flexion and extension of hip, knee, and ankle joints with the help of the physician and CPM, at least three times a day, at least 15 min each time
		(3) Asked frequently turn over, clap back
		(4) Acupuncture
		(5) Manipulation
		(6) Oral traditional Chinese medicine

#### Traditional care pathway

There was no detailed plan in the controlled group, and anti-infection, anti-coagulation, and other measures were taken to prevent complications during the perioperative period.

### Randomization and allocation

After the screening, patients will be randomized into two groups with an allocation ratio of 1:1. The randomization will be generated via SAS PROC PLAN software (SAS Inc., Cary, NC, USA) by an independent third-party clinical research organization (Institute of Basic Research in Clinical Medicine, China Academy of Chinese Medical Science) and concealed from the researchers by a senior data manager who is not involved in the study. The group assignment list will be sealed in opaque envelopes and be opened by the researchers following informed consent procedures and baseline testing.

### Blinding

All the investigators, physicians, nurses, assessors, analysts, and participants will be blinded to the group assignment until the end of the trial, when all statistical analyses are finished. If, after the first administration, any adverse event potentially related to the treatment occurs, the study physician will re-evaluate the participant, and PI will decide whether the non-blinded procedure is necessary. If non-blinding is required, the allocation information will be provided.

### Outcome measurements

#### Primary outcome measurement

LOS (length of stay) associated with ITF is a major public health issue due to the aging population, and it is the most objective outcome of evaluating the recovery pathway. Furthermore, high LOS would correspond with increases in postoperative complications [14, 19]. So we choose the LOS at discharge as the primary outcome measurement to assess the speed of recovery. Economic indicators including major medical expenses, bed expenses, drug expenses, inspection expenses, operation expenses, cost of anesthesia, nursing care, and blood transfusion are the other primary outcome measurement.

#### Secondary outcome measurements

Postoperative joint-specific function will be measured using the Harris score, which is widely used to assess the joint function of life for ITF patients. The Harris score is composed of 10 questions, 2 questions (ROM and absence of deformity) for the physician physical examination component and 8 questions for the patient-reported outcome component [20–24]. VAS is a reliable and valid measurement of pain. It has a horizontal, 100-mm-long line, with “no pain” recorded on the left side (score 0) and “pain as bad as it could be” on the right side (score 10) [25]. The other secondary outcomes included time to get out of bed, 30-day readmission rates, postoperative transfusion rates, discharge to home, and mortality.

### Safety assessments

Infection, deep venous thrombosis, and cardiovascular accidents will be recorded at each visit during treatment.

### Sample size calculation

We calculate the sample size according to our primary study. We conducted a preliminary clinical trial and pilot trial about ERAS care pathway versus traditional care pathway from January 2018 to May 2018. The primary outcome parameter was the LOS. Based on the previous results, we found that the primary outcome parameter of the ERAS care pathway group was 5.8 days and that of the traditional care pathway group was 9.2 days. According to the formula of the rate in completely random design, *n*_1_ = *n*_2_= $\frac{\left[{u}_{a/2}\sqrt{2}\overline{p}\left(1-\overline{p}\right)+{u}_{\beta}\sqrt{p_1\left(1-{p}_1\right)}+{p}_2\left(1-{p}_2\right)\right]}{{\left({p}_1-{p}_2\right)}^2}$, among which *n*_1_ and *n*_2_ were the number of patients in the two *u*_*a*/2_ = 1.96 when type 1 error is 0.05 and *u*_*β*_ = 1.282 when type II error is 0.1, respectively, in two-sided tests. $\overline{p}$ is the mean of *p*_2_ [26]. A two-sided 5% significance level and 90% power in detecting treatment differences were considered, and the above relevant data were input into SPSS 20.0 software. This number of patients actually provided less than 80% power, considering an estimated dropout rate of 20%. Therefore, we will recruit a total of 72 patients with 36 patients in each group.

### Statistical analyses

Prior to all analyses, a detailed statistical analysis protocol will be developed. All data will be analyzed in the clinical research center of Longhua Hospital affiliated to Shanghai University of TCM by statisticians blinded to allocation using the SPSS 20.0 statistical software (SPSS Inc., Chicago, IL). Efficacy and safety analyses will be conducted according to the intention-to-treat principle using the “last observation carried forward” rule. Before randomization, baseline characteristics will be collected as descriptive statistics for each patient, including gender, age, BMI, duration of symptoms, preoperative red blood cell count, and preoperative hemoglobin count. The data analysis of the primary outcome is based on the per-protocol population as a supportive analysis. Mean, standard deviation, median, quartiles and inter-quartiles for continuous variables, and frequency for categorical variables will be calculated. Continuous variable followed the normal distribution will be presented as means with standard deviations (SDs) and calculated by an independent sample Student’s *t* test; otherwise, the data will be expressed as medians with ranges, and non-parametric tests will be used. Categorical variables will be expressed as number (%) and analyzed by *χ*^2^ test or Fisher’s exact test. A *P* value of less than 0.05 is defined as statistically significant with two-sided 90% confidence intervals (CIs). Missing data will be input with the last observed response carried forward for all measures using the “last value carried forward” principle.

### Quality control

Prior to the clinical trial, we will carry out unified training to make sure all the physicians, nurses, and assessors involved fully understand the process of the trial, including selecting patient screening and selection, case report form writing, and manipulation details. To guarantee the quality of the whole trial, rigorous monitoring will be performed by three trained quality supervisors. During the trial, supervisors will check on case report forms and intervention twice a month. After verifying the case report forms, data will be input into the database by two full-time research members independently. The standard operating procedures (SOP) will be invariably followed. Dropouts, withdrawals (and the reasons), and any compliance of all patients occurring will be recorded in detail by the inspectors throughout the treatment and follow-up period.

## Discussion

The surgical stress response associated with major surgery describes fundamental metabolic changes that lead to increased catabolism, immunosuppression, free radical production, and hypercoagulable states. These physiologic alterations have been linked to changes in organ function resulting in undesirable postoperative morbidity, complications, pain, fatigue, and extended convalescence. ERAS attempts to decrease the surgical stress response to minimize postoperative complications and improve surgical outcomes and functional rehabilitation after major surgery.

ITF commonly occurred in the elderly and caused high mortality rate due to the complications of loss of walking ability. Due to the great importance of good functional outcome and avoidance of serious postoperative complications, the general consensus is that early surgery is the first choice of treatment for the elderly patients and PFNA is a mature surgical procedure.

Several clinical literatures describing ERAS on arthroplasty have been published previously in the literature, but there is a lack of prospective high-quality data from larger cohorts on it for improved recovery after surgery. In particular, we are unaware of published ERAS program on the field of ITF. Based on the theory of TCM, manipulations, acupuncture, and oral Chinese medicine are of great significance in promoting fracture healing and improving patients’ whole body state. With the objective, we have conducted a systematic literature search including Wan Fang Data, CNKI databases, Vip Journal Integration Platform, Chinese BioMedical databases, PubMed, MEDLINE, EMBASE, Cochrane Library, and ISI web of knowledge, and ERAS pathway integrated Chinese and western medicine was established by Delphi expert-opinion process including ten experts. The new ERAS project is consist of a multidisciplinary evidence-based approach to preoperative management, intraoperative control, and rehabilitation.

To our best knowledge, our study is the first elaborately designed, randomized, controlled trial to evaluate the clinical effect of the ERAS project integrated Chinese and western medicine on perioperative outcomes in ITF patients undergoing PFNA. A hallmark of ERAS is coordination between care services before and after surgery and continual evaluation of postoperative course with attention toward pain control, functional recovery, and patient satisfaction to improve standards of care. So the study is designed as a comprehensive study of LOS, economic indicators, pain relief, functional outcome, and complications. Outcome measurements are widely used in research to establish baselines, evaluate the effect of an intervention, and motivate patients’ self-evaluation. LOS was the most commonly assessed metric in comparative analyses. And a comparative reduction in LOS was reported in most studies using the ERAS protocol. Improved cost-effectiveness of surgical interventions is also a primary objective when implementing strategies for faster recovery in this study. The calculation of cost-effectiveness provided is based on data provided by our Department of Finance. The study is also to evaluate whether ERAS program can lead to a reduction in hospitalization costs, care costs, and nursing costs, which supports the cost-effectiveness of “fast-track” protocols in PFNA surgery. The VAS measurements, which have been found valid, reliable, and easy to apply in researches, are often used as the criterion standard to evaluate the pain intensity. The Harris score, a self-administered questionnaire, has been widely adopted as the criterion standard to estimate disease activity for its reliability and validity. Another aim of the study lies in reducing both intra- and postoperative adverse events and complication, which have the potential to impair the patients’ perioperative well-being and to prolong recovery. Especially in elderly patients, or in those with severe comorbidities, strict patient selection is key to both eligibility for safe ERAS and treatment in specialized short-stay clinics that may not have an ICU available. Administration of prophylactic medication against infections and thrombosis, prevention of hypothermia, and fluid imbalance, as well as operative measures, has therefore become integral to successful implementation of ERAS [27–30].

The lack of good quality RCTs in the field leaves us with notable gaps in our knowledge, and in clinical practice, many decisions have to be taken without the benefit of high-quality evidence. So we decide to conduct a prospective, randomized, controlled clinical trial, which also ensures the compliance of participants and meets ethical considerations, to closely detect the clinical efficacy of the ERAS program. The present study is built on our preliminary open experiment: a small sample-sized, randomized, and controlled trial of the ERAS program for patients with ITF. The result of the preliminary trial has shown that LOS of the ERAS care pathway group was 5.8 days and that of the traditional care pathway group was 9.2 days.

We hope that this trial, with a larger number of patients, can provide adequate statistical power to further analyze and explore the efficacy of ERAS. Thus, quality control is vital to the whole study, as we described in the protocol. To perform a reliable study, we will carry out unified training to make sure all the physicians, nurses, and assessors involved in the trials fully understand the process and details of ERAS program before the clinical trial. Hopefully, this trial will produce high-quality evidence pertaining to the efficacy and safety of the ERAS in treating patients with ITF. The results will aid in clinical decision-making for the management and provide useful information that can be incorporated into future guidelines.

## Data Availability

The datasets used and/or analyzed during the current study are available from the corresponding author on reasonable request. Ethics approval and consent to participate. The study design, procedures, and informed consent procedure were approved by the Longhua Hospital. Consent to participate will be obtained from the participants.

## Abbreviations

ERAS: Enhanced Recovery after Surgery
ITF: Intertrochanteric fracture
LOS: Length of stay
PFNA: Proximal femoral nail anti-rotation
TCM: Traditional Chinese medicine
